# Association of *HOTAIR* rs1899663 G>T Polymorphism with Colorectal Cancer in the Turkish Population: A Case–Control Study

**DOI:** 10.5152/tjg.2022.21836

**Published:** 2022-08-01

**Authors:** Berrin Yalınbaş Kaya, Betül Peker, Fuzuli Tuğrul, Özge Alkan Tali, Süleyman Bayram

**Affiliations:** 1 Department of Internal Diseases and Gastroenterology, Eskişehir City Hospital, Turkey; 2 Department of Pathology, Yunus Emre State Hospital, Eskişehir, Turkey; 3 Department of Radiation Oncology, Eskişehir City Hospital, Turkey; 4 Department of Internal Diseases, Eskişehir City Hospital, Turkey; 5 Department of Public Health Nursing, Adıyaman University Faculty of Health Sciences, Turkey

**Keywords:** Colorectal cancer, HOTAIR, rs1899663 polymorphism

## Abstract

**Background:**

Long noncoding RNAs increase the overexpression of oncogenes. Cancer development and metastasis of cancer may occur as a result of overexpression of oncogenes. Polymorphisms in the genes (such as *HOTAIR*, etc.) in which long noncoding RNAs are synthesized affect the expression of these genes. Therefore, these polymorphisms may play a role in cancer development and cancer metastasis. The present study aimed to evaluate the association between *HOTAIR* gene rs1899663 G>T polymorphism with colorectal cancer.

**Methods:**

The current study examined the *HOTAIR* gene rs1899663 G>T polymorphism in 100 patients with colorectal cancer and 93 healthy persons by a real-time polymerase chain reaction.

**Results:**

The G and T allele frequencies of the *HOTAIR* rs1899663 polymorphism were significantly different between the case and control groups (*P* = .01). The persons carrying the G allele had a protective effect against colorectal cancer, while individuals carrying the T allele were predisposed to colorectal cancer (*P* = .001). Four of 5 colorectal cancer recurrence patients had the TT genotype (*P* = .02).

**Conclusion:**

This research is the first to demonstrate the relationship between colorectal cancer and the *HOTAIR* gene rs1899663 polymorphism in the Turkish population, which is a Caucasian population. Our results suggest that the rs1899663 G allele has a protective role for colorectal cancer in the Turkish population. However, it would be appropriate to conduct this research with a larger sample to confirm this result in the Turkish population.

**Main Points**

- Colorectal cancer is a fatal disease of exactly unknown etiology; therefore, it is necessary to investigate host genetic factors in detail to understand the molecular pathogenesis of colorectal cancer.
- This is the first study to reveal the relationship of *HOTAIR* gene rs1899663 polymorphism with colorectal cancer in a Turkish population, which is a Caucasian population.
- *HOTAIR* gene rs1899663 G>T polymorphism has an important effect on the risk of colorectal cancer.

## Introduction

Colorectal cancer (CRC) is a common and deadly disease in Turkey and around the world. In 2020, while there were over 1.9 million new cases worldwide, 21 000 of these were detected in Turkey. In cancer ranking, Turkey ranks third in both sexes with a share of 9% in males and 9.1% in females.^1^ The risk factors for CRC are a familial predisposition, inflammatory bowel disease, colon polyps, cholecystectomy, diet, overweight, physical inactivity, smoking, and age. Additionally, pro-inflammatory bacteria such as fusobacterium, *Bacteroides fragilis*, and enteropathogenic *Escherichia coli* also affect the development of CRCs.^2^

Colorectal cancers can develop through genetic mutations (mutations in *KRAS*, *BRAF*, *PIK3CA*, *WNT*, *MAPK/PI3K*, *P53*, *HER2*, and *APC* genes), epigenetic changes, and long noncoding RNAs/micro RNAs (LncRNA/miRNAs). These factors contribute to the change of adenoma into carcinoma in the normal colonic epithelium.^3,4^ It has also been reported that chromosomal instability, microsatellite instability, and CpG island methylator phenotype play a role in the pathogenesis of CRC.^4^ Long noncoding RNAs are a large group of RNA molecules with over 200 nucleotide lengths. They are transcribed by RNA polymerase II, and most of their functions are not entirely understood.^5^ Long noncoding RNAs are involved in the epigenetic, transcriptional, and post-transcriptional stages and play a role in the etiology and pathophysiology of many cancers.^5-7^ Overexpression of LncRNAs from *HOTAIR*, *MALAT1*, *H19*, and *XIST* genes leads to upregulation of genes that have a critical role in the tumor process such as oncogenes involved in the MYC or WNT signaling pathway.^8^

*Hox transcript antisense intergenic RNA* (*HOTAIR*) is transcribed from the antisense sequence of the *HOXC* gene, which consists of 6 exons located on chromosome 12q13.13.^9^ Expression of *HOTAIR* has been reported to be irregular in breast, stomach, pancreatic, hepatocellular, lung, and CRCs.^10,11^ Therefore, it is considered effective in cancer development, progression, and metastasis. Though the mechanism of *HOTAIR* in the pathogenesis of CRC is still being investigated, there is no clear conclusion. Recent studies show that functional single-nucleotide polymorphisms (SNPs) in the *HOTAIR* gene are associated with the expression and function of this gene in many cancers including CRC.^9,10^ It has been reported that especially these SNPs (rs920778, rs4759314, rs1899663, rs12826786, etc.) found in *HOTAIR* act as cancer susceptibility loci in lung cancer, gastric cancer, breast cancer, and neuroblastoma, and they are highly associated with increased risk of these cancers.^11^

There are not enough studies demonstrating the association of the *HOTAIR* rs1899663 G>T polymorphism, especially with CRC, and this association has never been surveyed in the Caucasian population.^8-11^ Therefore, the effects of *HOTAIR* rs1899663 G>T polymorphism on the CRC were investigated by taking 100 patients with CRC and 93 healthy persons in the Turkish population.

## Materials and Methods

This study was approved by the Osmangazi University Faculty of Medicine Ethics Committee and it was done according to the declaration of Helsinki. Our study was carried out at Eskişehir Yunus Emre State Hospital and Adıyaman University Faculty of Arts and Sciences Biology Department Research Laboratory. The individuals included in the study were informed in detail about the study, and ethics committee approval was obtained. Between 2018 and 2020, 100 patients who had CRC surgery in Eskişehir Yunus Emre State Hospital or who had multiple biopsies from cancerous tissue with the diagnosis of CRC in colonoscopy and evaluated in the pathology department were included in the study. All patients diagnosed with colon adenocarcinoma were included in our study. A total of 100 patients with colorectal cancer and 93 age- and sex-matched healthy individuals were included in the study. We obtained patients’ information including age, sex, family history of cancer, laboratory data, tumor localization (rectum, left colon, right colon, sigmoid colon, transverse colon, caecum), tumor diameter, recurrence, complete resection, tumor differentiation (well, moderate, and poor), ECOG (Eastern Cooperative Oncology Group) performance status, retrospectively. Tumor staging of CRCs was done using the Classification of Malignant Tumors (TNM), which was created and updated by the American Joint Committee on Cancer and the Union for International Cancer Control. Healthy volunteers were selected from those who applied to Eskişehir Yunus Emre State Hospital for checkup. Colonoscopy procedure was also performed on healthy volunteers, their biochemical parameters were also checked, and those who were confirmed to be healthy were included in the study.

### DNA Isolation

Tumor tissue samples were taken from suspicious surgical areas and fixed in 10% buffered formalin. Tumor samples were processed in an automated tissue-tracking device. The samples were embedded in paraffin to form paraffin blocks. After staining with hematoxylin and eosin, the sections were prepared from paraffin blocks.

Researchers who performed laboratory analysis were blinded to the identification and status of subjects because whole blood specimens of healthy controls and formalin-fixed, paraffin-embedded tissue sections of CRC patients were handled and made anonymous according to the ethical and legal standard. Whole blood specimens of healthy controls were drawn in a Vacutainer tube containing ethylene diamine tetraacetic acid and frozen at −80°C until genomic DNA (gDNA) was isolated. Genomic DNA was isolated from peripheral whole blood mononuclear cells of healthy controls and tumor tissue samples of CRC patients by the High Pure PCR Template Preparation Kit (Roche Diagnostics GmbH, Mannheim, Germany) in accordance with the manufacturer’s directions. The quantity and quality of gDNA were evaluated by the Qubit® fluorometer (Invitrogen, Carlsbad, Calif, USA).

### Genotyping of *HOTAIR* rs1899663 G>T Polymorphism

Genotyping of HOTAIR rs1899663 G>T polymorphism was carried out by using the commercially available TaqMan allelic discrimination assay 4351379 C–2104251_20 (Thermo Fisher Scientific Inc., Waltham, Mass, USA) according to the manufacturer’s directions. Real-time PCRs were done in the LightCycler 96 real-time PCR (Roche Diagnostics GmbH, Mannheim, Germany) according to the standard cycling conditions: 95°C for 10 minutes, followed by 40 cycles of 95°C for 15 seconds, and 60°C for 1 minute. Genotype calling for each subject was determined automatically by the LightCycler Genotyping software (Roche Diagnostics GmbH, Mannheim, Germany). Polymerase chain reaction was carried out in a total volume of 10 μL containing 5 μL 2× TaqMan® Universal Master Mix II (Thermo Fisher Scientific Inc., Waltham, Mass, USA), 900 nM of each primer (Thermo Fisher Scientific Inc.), 200 nM of each probe (Thermo Fisher Scientific Inc.), and approximately 10 ng gDNA. The context sequence (VIC/FAM) was (written 5′ → 3′) TCCAAAAGCCTCTAATTGTTGTCAC[A/C]TCCACCCTCCTCAACTGGAAAAATG.

### Statistical Analysis

The sufficient sample size and 80% statistic power were calculated with Quanto software (https://pphs.usc.edu/download-quanto/) using minor allele frequencies of rs1899663 polymorphism in HapMap (www.ncbi.nlm.nih.gov/snp/?term=rs1899663), the prevalence of the disease in the population, and the effect of minor allele on phenotype. All analyses were done by using the IBM Statistical Package for the Social Sciences 20 software (IBM Corp.; Armonk, NY, USA). Comparisons in demographical variables between both groups were done using the independent samples *t*-test or Mann–Whitney *U* test for continuous variables and chi-square test for categorical variables. Regression analysis with adjustment for age and sex was used to establish risk determinants in statistical models between the groups. Analyses were two-sided. *P* < .05 were accepted statistically significant.

## Results

Demographic and clinicopathological features of CRC patients and control subjects are shown in Table 1. There was no significant difference observed between the case and control groups by age (*P* = .14) and sex (*P* = .13). Predominantly, tumor localization of 100 CRC cases was determined as 31% sigmoid colon, 27% rectum, and 22% cecum. Tumor size was >4 cm in 48% of the cases, while tumor size was ≤4 cm in 52% of the cases. In total, 21% of the patients had distance metastasis and 79% of the patients did not have distance metastasis. Tumor resection was successfully performed in 97 of 100 CRC patients, and tumor recurrence was found in 5, while it was not found in 80 of the patients during the follow-up.

**The Relationship Between CRC Risk and HOTAIR rs1899663 G>T Polymorphism**

Table 2 shows the association of HOTAIR rs1899663 G>T polymorphism with the risk of susceptibility to CRC according to different inheritance models. The G and T allelic frequencies of the *HOTAIR* rs1899663 polymorphism (53% and 47% in CRC patients and 66% and 34% in controls, *P* = .01, respectively) were significantly different between case and control groups. Moreover, while the *HOTAIR* rs1899663 G>T polymorphism was statistically significant in codominant, dominant, and recessive genetic models for CRC risk, it was not statistically significant in the overdominant genetic model (Table 2). In the allele and genotype frequency analysis between the 2 groups, it was determined that the persons carrying the G allele had a protective effect against CRV, while individuals carrying the T allele were predisposed to CRC (for the T vs G alleles, odds ratio (OR) = 0.59, *P* = .01; for the TT vs GG genotypes, OR = 0.24, *P* = .001) (Table 2).

As shown in Table 3, the relationship between clinicopathological characteristics of the cases and *HOTAIR* rs1899663 G>T polymorphism was evaluated by stratification analysis. When the clinicopathological variables were categorized as tumor size (≤4 cm and >4 cm), ECOG performance status (0 + I and II + III), tumor differentiation (well and moderate + poor), family history of cancer (yes and no), TNM stage (T1 + T2 and T3 + T4; N0 and N1 + N2; M0 and M1), and the CRC recurrence, the CRC recurrence highly reached significant a *P* value, except for others variables (Table 3). The 4 of 5 CRC recurrence patients had the TT genotype (TT vs TG + GG, *P* = .02).

## Discussion

Colorectal cancer is one of the main causes of death from neoplasia. Familial predisposition, carcinogen molecules, and genetic factors have a vital role in the development of CRC. During embryological development, the HoxC genes encode transcription factors for the anterior-posterior axis of the intestine.^12-14^ Long noncoding RNAs, which are transcribed from the antisense sequence of the *HOXC* gene, have an important role in the cells’ system such as chromatin remodeling, epigenetic arrangement, gene silencing, regulation of gene expression, etc.^6,8^ The association of overexpression of *HOTAIR* with tumor cell invasion, recurrence, and poor prognosis in some malignant tumors has still been under investigation. Studies have suggested that SNPs of *HOTAIR* may be associated with an increased risk of various cancers.^15,16^ In the studies conducted in the Turkish population, *HOTAIR* rs12826786, rs1899663, and rs920778 polymorphisms were investigated in gastric, lung, and breast cancer. *HOTAIR* polymorphisms were not associated with gastric cancer, but it was suggested that they could play an important role in genetic susceptibility to lung and breast cancer development and invasion.^17-19^ Our study is the first to investigate the association of *HOTAIR* rs1899663 polymorphism with CRC in the Turkish population.

In the current study, we investigated the association between *HOTAIR* rs1899663 G>T and CRC. There was a relation between the rs1899663 polymorphism’s T allele and susceptibility to CRC. Our data suggest that the G allele (T vs G; OR = 0.59; *P* = .01; for TT vs TG + GG: OR = 0.47; *P* = .013) and GG genotype have a protective role (for TT vs TG, OR = 0.36; *P* = .002; for TT vs GG, OR = 0.24; *P* = .001) (Table 2). The results presented in Table 2 show that the rs1899663 G>T polymorphism has a significant effect on the development of CRC. There are few studies to investigate the relationship between CRC and *HOTAIR* polymorphisms, but there is only one study conducted with the rs1899663 variant in the CRC. That study was conducted on the Asians,^8^ and the association of this polymorphism with CRC has never been investigated in the Caucasian population. Similar to our findings, Kim et al^8^ declared that the rs1899663 G>T polymorphism’s TT genotype was associated with an increase in colon cancer mortality and the GT genotype increased the risk of CRC in the Korean population.^8^

In the meta-analysis reported by Zhang et al^9^ the rs1899663 variant was analyzed in 3 of 26 studies, and no significant correlation was found with the cancer risk including breast, esophagus, and stomach cancers. Liu et al^16^ reported that in a meta-analysis of 17 of 116 studies including a total of 17 048 subjects, some polymorphisms including rs1899663 in *HOTAIR* play a role in genetic predisposition to other cancers in recessive and homozygote models in the Asian population. Our findings confirm the results of this meta-analysis, rs1899663 G>T polymorphism’s T allele was determined as the risk allele.

Contrary to our findings, in the 6 different cancers studied with 3239 cases and 4067 controls, rs1899663 polymorphism was not significant in the Asian population.^13^ Similarly, Xu et al^20^ reported that rs1899663 was explored for the relationship with cancer risk (except for CRC) in a meta-analysis including 9 studies, but it was not found to be significant.^20^ In studies conducted on the association between different cancers with this polymorphism, it has been reported that the rs1899663 T allele increases the risk of cancer in breast, lung, ovarian, and prostate cancers in different populations.^10,15,21,22^ However, Ren et al^23^ declared that rs1899663 has no significance in lung cancer. Recently, a meta-analysis published by Li et al^24^ indicates that rs920778, rs4759314, rs874945, and rs12826786 polymorphisms significantly increased with susceptibility to overall cancer. However, rs7958904 and rs1899663 under any 5 genetic models have no impact on susceptibility to overall cancer.^24^ Furthermore, altered cancer risk was detected when the data were stratified by cancer type, ethnicity, the source of controls, and Hardy–Weinberg equilibrium (HWE) in all the SNPs. It should be noted that the different meta-analyses carried out show differences in terms of ethnicity, type of cancer, and even the method of analysis (Restriction Fragment Length Polymorphism [RFLP] or Taqman).^16,20,24^ Therefore, it would be very important to continue analyzing the role of this polymorphism in CRC in different populations.

This study also investigated the association between this polymorphism and clinicopathological features of patients with CRC; 4 of 5 recurrence patients had rs1899663 TT genotype, and it was statistically significant in tumor recurrence (for TT vs TG + GG: *χ* ^2^ = 5.33; *P* = .02). No statistically significant correlation was found between rs1899663 polymorphism and other clinicopathological features (ECOG performance status, differentiation, T stage, N stage, distant metastasis, tumor size, and family history of cancer). In CRC, there is no other study that analyzes the relationship between clinicopathological features and this polymorphism, and so it is not possible to make a comparison.

Several possible limitations in our study required to be addressed are as follows: (a) All participants included in the current study were from Eskişehir and surrounding provinces, whence, the present work does not represent all patients with CRC in the Caucasian population. (b) The sample size of the present study may not have statistical power to define a small impact from low penetrating genes or SNPs. Notwithstanding this limitation, we had enough statistical power to reveal the influence of rs1899663 G>T polymorphism in *HOTAIR* gene on CRC risk. But, further studies with larger sample sizes are needed to verify our present observation in a Caucasian population. (c) This study only investigated the relationship of HOTAIR rs1899663 polymorphism with CRC. Therefore, the relationship of other *HOTAIR* polymorphisms with CRC has not been established.

In conclusion, this research is the first to demonstrate the relationship between CRC and the *HOTAIR* gene rs1899663 G>T polymorphism in the Turkish population, which is a Caucasian population. Similar to our findings, the rs1899663 T allele is a risk factor for CRC in the Asian population, and our results suggest that the rs1899663 G allele has a protective role (on the other hand, T allele is a risk factor) for CRC in the Turkish population. However, it would be appropriate to conduct this research with a larger sample to confirm this result in the Caucasian population.

## Figures and Tables

**Table 1.** Clinical Characteristics of Colorectal Cancer Cases and Control Subjects Enrolled in the Study

Characteristic	Colorectal Cancer (n = 100)	Controls (n = 93)	*P*
Age (year, mean ± SD)	64.46 ± 11.07	61.77±12.00	.14
Sex			.13
Males	74 (67.3%)	67 (72.0%)	
Females	26 (31.3%)	26 (28.0%)	
Tumor localization (%)			
Rectum	27 (27.0%)		
Colon			
Left colon	4 (4 %)		
Right colon	10 (10 %)		
Sigmoid colon	31 (31 %)		
Transverse colon	6 (6 %)		
Cecum	22 (22 %)		
Hb (g/dL) (mean ± SD)	12.07 ± 2.45		
Alanine transaminase (ALT) (IU/L) (mean ± SD)	16.96 ± 12.38		
Aspartate transaminase (AST) (IU/L) (mean ± SD)	20.42 ± 11.45		
Na (mmol/L) (mean ± SD)	138.69 ± 3.00		
K (mmol/L) (mean ± SD)	4.257 ± 0.52		
CEA (ng/mL) (mean ± SD)	27.75 ± 1.72		
CA 19-9 (U/mL) (mean ± SD)	50.52 ± 1.81		
ECOG performance status			
0	51 (58.0%)		
I	31 (35.2%)		
II	5 (5.7%)		
III	1 (1.1%)		
Differentiation			
Well	41 (41.0%)		
Moderate	52 (52.0%)		
Poor	7 (7.0%)		
T stage			
T1	6 (6.0%)		
T2	11 (11.0%)		
T3	62 (62.0%)		
T4	21 (21.0%)		
N stage			
N0	56 (56.0%)		
N1	28 (28.0%)		
N2	16 (16.0%)		
Distance metastasis			
M0 (absent)	79 (79.0%)		
M1 (present)	21 (21.0%)		
Tumor size (cm)			
≤4 cm	48 (48.0%)		
>4 cm	52 (52.0%)		
Resection			
R0	97 (97.0%)		
R1	3 (3.0%)		
Recurrence			
Absent	80 (80.0%)		
Present	5 (5.0%)		
Unknown	15 (15.0%)		
Chemotherapy			
Absent	32 (32.0%)		
Present	55 (55.0%)		
Neo-Adjuvant	8 (8.0%)		
Unknown	5 (5.0%)		
Family history of cancer			
Yes	49 (49.0%)		
No	51 (51.0%)		

SD, standard deviation.

**Table 2.** Allele and Genotype Frequencies in the Colorectal Cancer Cases and the Healthy Control Groups As Well As the Association of *HOTAIR* rs1899663 T>G Polymorphism with the Risk of Colorectal Cancer Susceptibility According to Different Models of Inheritance

	Controls n = 93 (%)	Colorectal cancer n = 100 (%)	OR (95% CI)	*P* ^a^	AIC	BIC
**rs1899663**						
Allele						
T	64 (34.0)	94 (47.0)	1.00 (Reference)			
G	122 (66.0)	106 (53.0)	0.59 (0.39-0.89)	0.01		
Codominant						
TT	12 (12.9)	33 (33.0)	1.00 (Reference)		260.5	270.2
TG	40 (43.0)	40 (40.0)	0.36 (0.16-0.80)	0.002		
GG	41 (44.1)	27 (27.0)	0.24 (0.11-0.54)	0.001		
Dominant						
TT	12 (12.9)	33 (33.0)	1.00 (Reference)		260	266.6
TG + GG	81 (87.1)	67 (67.0)	0.30 (0.14-0.63)	<0.001		
Recessive						
TT + TG	52 (55.9)	73 (73.0)	1.00 (Reference)		265.1	271.6
GG	41 (44.1)	27 (27.0)	0.47 (0.26-0.86)	0.013		
Overdominant						
TT + TG	53 (57.0)	60 (57.5)	1.00 (Reference)		271.1	277.6
GG	40 (43.0)	40 (42.5)	0.88 (0.50-1.57)	0.67		
Log-additive	--	--	0.51 (0.34-0.76)	<0.001	259.4	265.9

^a^Data were calculated by logistic regression analysis.

AIC, Akaike’s information criterion; BIC, Bayesian information criterion.

**Table 3.** Genotype Distribution of *HOTAIR* rs1899663 T>G Polymorphism Concerning Clinicopathological Features of Colorectal Cancer Patients

Variables		*HOTAIR* rs1899663 T>G Polymorphism		Total	χ^2^	*P*
TT	TG + GG
ECOG performance status	0 + I	18 (35.3%)	33 (64.7%)	51	0.30	.58
II + III	11 (29.7%)	26 (70.3%)	37
Differentiation	Well	13 (31.7%)	28 (68.3%)	41	0.05	.82
	Moderate + poor	20 (33.9%)	39 (66.1%)	59
T stage	T1 + T2	6 (35.3%)	11 (64.7%)	17	0.05	.83
	T3 + T4	27 (32.5%)	56 (67.5%)	83
N stage	N0	18 (32.1%)	38 (67.9%)	56	0.04	.84
	N1+N2	15 (34.1%)	29 (65.9%)	44
Distant metastasis	M0 (absent)	25 (31.6%)	54 (68.4%)	79	0.31	.58
	M1 (present)	8 (38.1%)	13 (61.9%)	21
Tumor size (cm)	≤4 cm	15 (31.2%)	33 (68.8%)	48	0.13	.72
	>4 cm	18 (34.6%)	34 (65.4%)	52
Recurrence	Absent	24 (30.0%)	56 (70.0%)	80	5.33	**.02**
	Present	4 (80.0%)	1 (20.0%)	5
Family history of cancer	Yes	14 (27.5%)	37 (72.5%)	51	1.45	.23
	No	19 (38.8%)	30 (61.2% )	49
